# Catheter removal and outcomes of multidrug-resistant central-line-associated bloodstream infection

**DOI:** 10.1097/MD.0000000000012782

**Published:** 2018-10-19

**Authors:** Jason P. Burnham, Rebecca P. Rojek, Marin H. Kollef

**Affiliations:** a Division of Infectious Diseases, Washington University School of Medicine; b Barnes-Jewish Hospital; c Division of Pulmonary and Critical Care Medicine, Washington University School of Medicine, St Louis, MO.

**Keywords:** central-line-associated bloodstream infection, multidrug-resistant organisms

## Abstract

Central-line-associated bloodstream infections (CLABSIs) are responsible for ∼1/3 of all deaths from healthcare-associated infections in the United States. Of these, multidrug-resistant organisms (MDROs) are responsible for 20% to 67%. However, whether catheter removal affects clinical outcomes for MDRO CLABSIs has not been studied. Our objective was to determine the relationship between failure to remove a central venous catheter (CVC) and 30-day all-cause mortality in patients with MDRO CLABSIs. We used a retrospective cohort from Barnes-Jewish Hospital (1/1/2009–10/1/2015) to study patients with a multidrug-resistant *Staphylococcus aureus*, *Enterococcus* species, *Enterobacteriaceae*, *Acinetobacter* species, or *Pseudomonas aeruginosa* CLABSI. Risk factors for 30-day mortality, including catheter removal, were assessed for association with 30-day mortality using Cox proportional hazards models. The CLABSIs were assessed prospectively at the time of occurrence by infection prevention specialists. A total of 430 patients met inclusion criteria, 173 (40.2%) with *Enterococcus*, 116 (27.0%) *Enterobacteriaceae*, 81 (18.8%) *S aureus*, 44 (10.2%) polymicrobial, 11 (2.6%) *P aeruginosa*, and 5 (1.2%) *Acinetobacter* CLABSIs. Removal of a CVC occurred in 50.2% of patients, of which 4.2% died by 30 days (n = 9). For patients whose CVC remained in place, 45.3% died (n = 97). Failure to remove a CVC was strongly associated with 30-day all-cause mortality with a hazard ratio of 13.5 (6.8–26.7), *P* < .001. Other risk factors for 30-day mortality included patient comorbidities (cardiovascular disease, congestive heart failure, cirrhosis), and being in an intensive care unit at the time of MDRO isolation. Failure to remove a CVC was strongly associated with 30-day all-cause mortality for patients with MDRO CLABSIs in this single center retrospective cohort. This suggests that patients presenting with MDRO CLABSIs should all undergo CVC removal.

## 1 Introduction

Central-line-associated bloodstream infections (CLABSIs) are one of the most common causes of healthcare-associated infections (HAIs). There are >20,000 CLABSIs each year in the United States and significantly more worldwide.^[1,2]^ In addition, CLABSIs are responsible for one-third of all deaths from HAIs in the United States and associated with billions of dollars in excess costs.^[3,4]^ Guidelines for treatment of CLABSIs have been developed that recommend central line removal in many situations,^[5]^ but they did not specifically consider the management of multidrug-resistant organism (MDRO) CLABSIs. At present, guidelines suggest that for enterococcal and gram-negative bacillus CLABSIs, retaining the central venous catheter (CVC) is an option (if antibiotic-lock therapy is used concurrently).^[5]^

As MDROs are more difficult to treat,^[6]^ source control with catheter removal would, in theory, be more critical for patients with MDRO CLABSIs, but this has not yet been studied. *Staphylococcus aureus*, *Enterobacteriacaeae*, *Pseudomonas aeruginosa*, *Enterococcus*, and *Acinetobacter* make up over half of all CLABSI pathogens and are drug-resistant threats listed by the Centers for Disease Control and Prevention (CDC).^[6–9]^ MDROs are responsible for 20% to 67% of all CLABSIs, making it critical to understand the best management strategy for these patients.^[8,10]^ Therefore, it was our goal to determine the relationship between failure to remove a CVC and 30-day all-cause mortality in patients with MDRO CLABSIs. We utilized multiple definitions of drug resistance as outlined by the CDC and European CDC (Supplemental Table 1).^[11–13]^ Understanding the association between central-line removal and mortality will help guide catheter removal decisions for patients with MDRO CLABSIs.

## 2 Materials and methods

### 2.1 Study location and patient population

This study was conducted at Barnes-Jewish Hospital, a 1250 bed academic medical center located in St Louis, MO. The study period was January 1, 2009 to October 1, 2015. All hospitalized patients with a positive blood culture for *Enterobacteriaceae*, *Enterococcus* spp, *S. aureus*, *P aeruginosa*, or *Acinetobacter* spp. were analyzed for eligibility. Organisms were identified using culture and Matrix Assisted Laser Desorption-Ionization Time of Flight Mass Spectrometry with Vitek MS v2.0 software (bioMerieux, Durham, NC). Antimicrobial susceptibilities for all pathogens were determined using disc diffusion methodology. Patients were excluded if they had drug-susceptible pathogens or died or were discharged within 24 hours after the first positive culture was collected. This study was approved by the Washington University School of Medicine Institutional Review Board with a waiver of informed consent.

### 2.2 Study design and data collection

Utilizing a retrospective cohort study design, the first hospitalization between January 2009 and October 2015 of all patients age ≥18 with MDR *Enterobacteriaceae*, *Enterococcus* spp, *S aureus*, *P aeruginosa*, or *Acinetobacter* spp. isolated from blood culture were identified. Beginning in 2009, infection prevention (IP) specialists reviewed all blood cultures to determine if patients had a CLABSI. Based on the determination of the IP specialists at the time of the cultures, we excluded all patients who were determined not to have a CLABSI. All CLABSI determinations were made based on National Healthcare Safety Network definitions in use at the time the culture was drawn.^[14]^ The primary endpoint was 30-day all-cause mortality. Baseline characteristics, including age, gender, race, Acute Physiology and Chronic Health Evaluation (APACHE) II scores (calculated based on clinical data from the 24 hours before and after positive cultures were obtained), and medical comorbidities (based on ICD-9-CM diagnosis codes) were obtained. Patients who died during the index hospitalization or were discharged on hospice were considered to be expired at the time of hospital discharge. Over 50% of patients entering hospice care die within 3 weeks^[15]^ and often cannot receive intravenous antibiotic therapy. In addition, the majority of patients entering hospice care do not recover to the point of being discharged alive from hospice care.^[16]^ Therefore, we considered the expiration date for patients discharged on hospice to be the date of hospital discharge.

### 2.3 Definitions and data sources

Time to death was calculated from the day that a positive culture qualifying for a MDRO CLABSI was obtained. All data were obtained from the BJC Healthcare Informatics database, maintained by the Center for Clinical Excellence, BJC Healthcare. Expiration dates from stays in any BJC facility are included in the Informatics database. Patients without follow up in the BJC system and not in the Social Security Death Index (SSDI) were considered lost to follow-up on their last date of care in a BJC facility (a total of 18 patients, 4.2% of the cohort).

#### 2.3.1 Defining MDROs

We utilized multiple definitions of drug resistance as outlined by the CDC and European CDC (Supplemental Table 1).^[11–13]^ Any *Enterobacteriaceae* was presumed to be an extended-spectrum beta lactamase producer if ceftriaxone or ceftazidime was intermediate or resistant. Patients were considered to have a vancomycin intermediate *S aureus* infection if *S aureus* isolated in culture was determined to have a vancomycin MIC of 4 or 8 μg/mL, in accordance with Clinical & Laboratory Standards Institute recommendations.^[17]^

#### 2.3.2 Central lines

We included all patients with short-term and long-term CVCs. Catheter removal dates were confirmed by retrospective chart review, including chest X-ray visualization when electronic medical records were insufficient to make a removal date determination. For patients where the central line was removed at or in the 24 hours before positive culture, lines were considered to be removed at 0 hours.

### 2.4 Statistical analysis

Comparisons between survivors and nonsurvivors were performed using the Chi-squred or the Fisher exact test for categorical values and Student *t* test or Mann–Whitney *U* test for continuous variables. Continuous variables were reported as means with standard deviations or medians and interquartile ranges (IQRs). Categorical data were expressed as frequencies. Kaplan–Meier curves were generated to graphically demonstrate the relationship between catheter removal and 30-day all-cause mortality, with log-rank test for significance. Multivariate Cox proportional hazards models were used to determine risk factors for 30-day mortality. The proportional hazards assumption was checked graphically using a log-log survival plot. A *P* value of <.05 was considered significant in all analyses. Factors associated with mortality in bivariate analysis (*P* < .10) were entered into a backward stepwise Cox proportional hazards model to determine hazard ratios (HRs) for death. In addition, factors associated with CVC removal (*P* < .2) were entered into the Cox proportional hazards model for death. All variables entered into the model were assessed for collinearity. For patients who underwent CVC removal, we used Mann–Whitney *U* tests to compare time to removal as a risk factor for death. All analyses were conducted with SPSS version 25 software (IBM, Armonk, NY).

## 3 Results

A total of 430 patients with MDRO CLABSIs were identified, 173 (40.2%) with *Enterococcus*, 116 (27.0%) *Enterobacteriaceae*, 81 (18.8%) *S aureus*, 44 (10.2%) polymicrobial, 11 (2.6%) *P aeruginosa*, and 5 (1.2%) *Acinetobacter*. Among patients with enterococcal CLABSIs, 90.2% (n = 156) were due to *Enterococcus faecium* and 9.8% (n = 17) due to *E. faecalis*. Among patients with *Enterobacteriaceae* CLABSIs, the majority were due to *Enterobacter cloacae* at 46.6% (n = 54) and *Escherichia coli* 29.3% (n = 34). The remaining were due to *Klebsiella aerogenes* (n = 10), *K. pneumoniae* (n = 6), *Citrobacter freundii* (n = 5), other *Enterobacter* species (n = 3), and *Proteus mirabilis* (n = 2). Among patients with polymicrobial MDRO infections, 15.9% (n = 7) were all gram-positive, 11.4% (n = 5) were all gram-negative, and 72.7% (n = 32) were mixed gram-positive/gram-negative infections.

**Figure 1:**
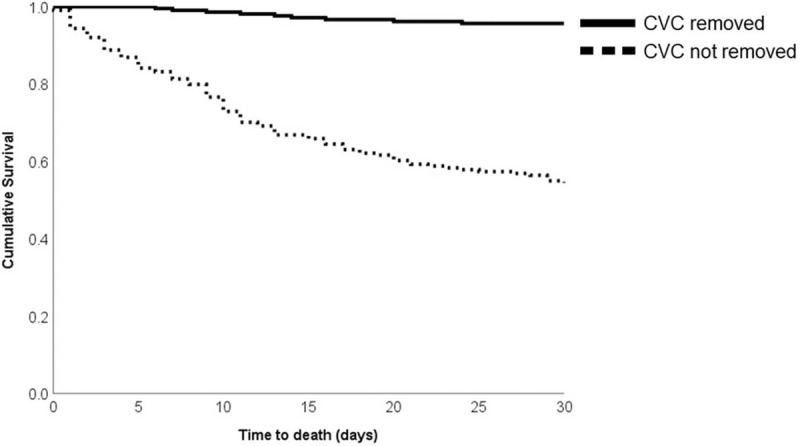
Kaplan–Meier curve for time to death with and without central venous catheter (CVC) removal.

The CVC removal occurred in 50.2% of patients, of which 4.2% died within 30 days (n = 9) of culture. Patients who did not have their CVC removed had lower APACHE II scores, were more likely to have a bone marrow transplant or lymphoma or have been in the intensive care unit (ICU) at the time of the MDRO CLABSI, and were less likely to have end-stage renal disease or paralysis (Supplemental Table 2). For patients whose CVC remained in place, 45.3% (n = 97) died within 30 days of culture. Failure to remove a CVC was significantly associated with 30-day all-cause mortality (Fig. 1). Among patients with gram-positive MDRO CLABSIs that did not have catheters removed, 30-day mortality was 54.1%; mortality was 5.0% for those who had their catheters removed. Analogously, for gram-negative MDRO CLABSIs that did not have catheters removed, 30-day mortality was 32.9%; mortality was 1.6% for those who had their catheters removed. Within 30 days, 8 patients had recurrent MDRO infections, 6/8 with organisms in the same class as the initial infection, 3 *Enterobacteriaceae,* 1 each of *Pseudomonas*, *Enterococcus*, and *S aureus*. The remaining 2 patients had different initial and subsequent MDRO infections.

Among patients who underwent CVC removal, the time to CVC removal was not significantly different between survivors and nonsurvivors (*P* = .94). In survivors, the median time to removal was 4.4 IQR (2.4–8.7) days and 4.8 (2.3–6.9) days in nonsurvivors.

In bivariate analysis, nonsurvivors were less likely to have their CVC removed or have MDR *Enterobacteriaceae* CLABSIs, more likely to have a bone marrow transplant, cardiovascular disease, congestive heart failure (CHF), chronic respiratory failure, cirrhosis, metastatic cancer, be in the ICU at the time of the MDRO CLABSI, or have *Enterococcus* CLABSIs (Table 1). After adjustment using a multivariate Cox proportional hazards model, failure to remove a CVC was strongly associated with 30-day all-cause mortality with a hazard ratio of 13.5 (6.8–26.7), *P* < .001. Other risk factors for 30-day mortality included patient comorbidities (cardiovascular disease, CHF, cirrhosis), and being in an intensive care unit at the time of the MDRO CLABSI (see Table 2).

**Table 1:**
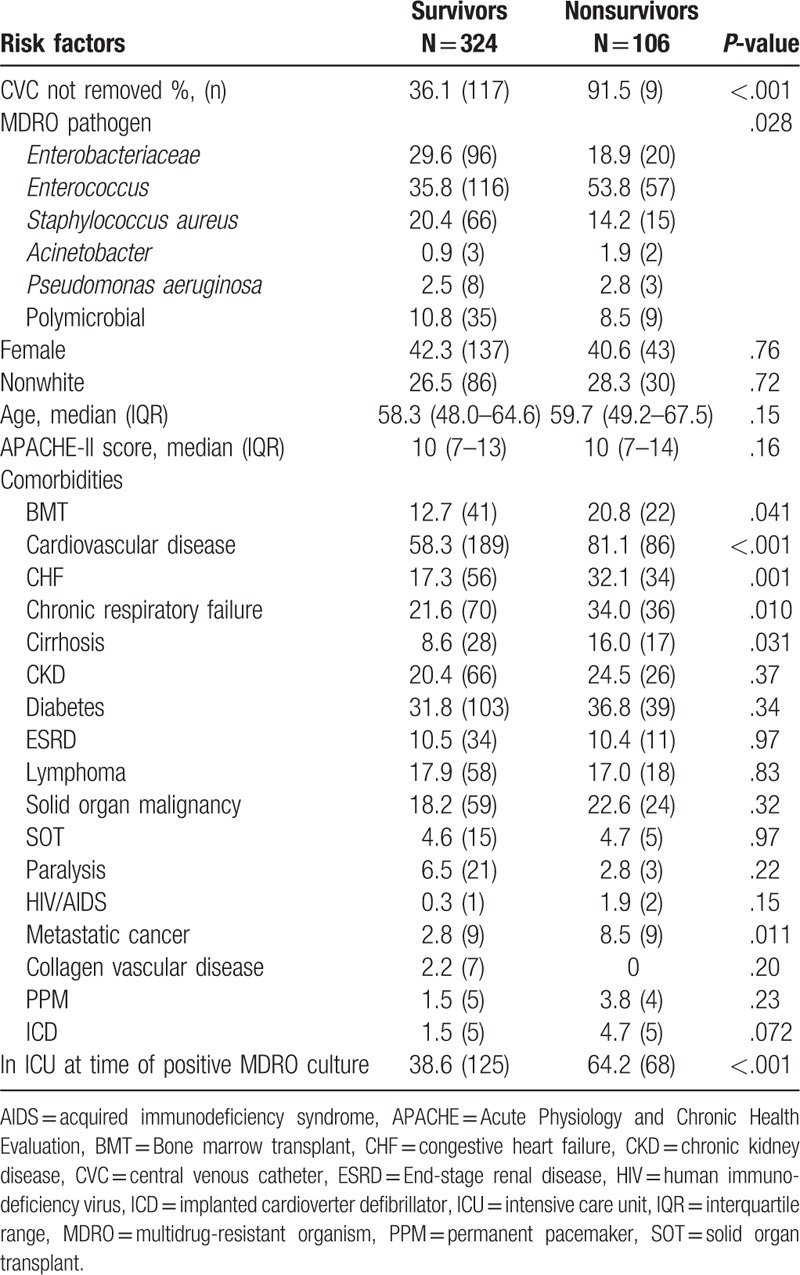
Univariate comparisons of risk factors for death between 30-day survivors and nonsurvivors.

**Table 2:**
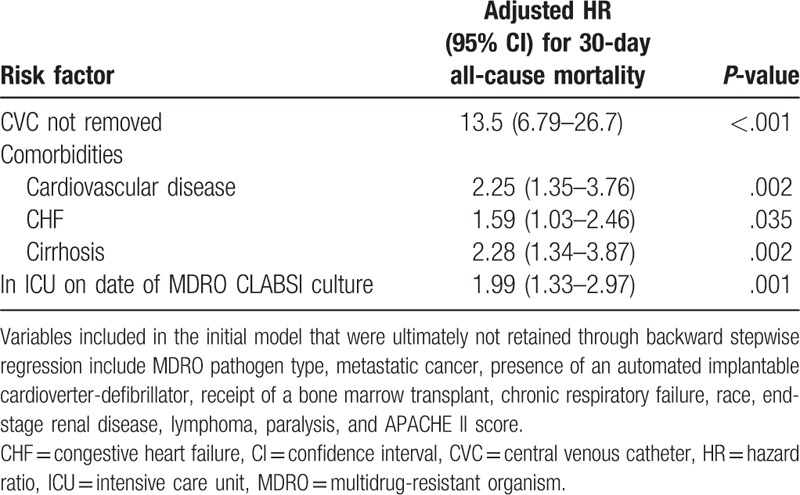
Risk factors for 30-day all-cause mortality from backward stepwise Cox proportional hazards model from cohort of patients with MDRO CLABSI.

## 4 Discussion

Infections due to MDROs cause more than 2 million infections and 23,000 deaths/year in the United States,^[6]^ and their incidence is on the rise globally.^[6,18,19]^ In addition, MDRO infections are associated with a 2- to 3-fold increase in hospital costs, a >12 days increase in hospital length of stay,^[20]^ 30-day readmission rates exceeding 30%,^[21]^ and 1-year mortality of 35% to 60%.^[22]^ Therefore, identifying factors that can improve outcomes for patients with these difficult to treat infections are critical. In this study, we identify CVC removal as a possible method to improve outcomes in patients with MDRO CLABSIs. Among CLABSIs, MDROs account for 20% to 67% of cases, reinforcing the importance of understanding best management strategies for these infections.^[8,10]^

We found that CVC removal was associated with decreased 30-day all-cause mortality for patients with various MDRO CLABSIs. Previous studies have shown that failure to remove a CVC is associated with an increased risk of hematogenous complications as well as mortality.^[23,24]^ In one study of gram-negative CLABSIs,^[25]^ there was a strong association between failure to remove a CVC and mortality. In addition, this study included patients with MDR gram-negative CLABSIs and found that CVC removal was particularly helpful in reducing mortality in this small sub group. We confirm these findings in a larger group of MDRO CLABSI patients and with a broader range of pathogens.

Among MDRO CLABSI patients that had their CVC removed, we did not find an association between the timing of catheter removal and mortality. Previous studies have found mixed results regarding timing of catheter removal and mortality.^[25,26]^ Given the large burden of unremoved CVCs in our study, it seems prudent to remove a CVC once a MDRO CLABSI has been identified as a first step in improving patient outcomes. Future studies with larger sample sizes can help clarify what role early CVC removal has in impacting clinical outcomes of MDRO CLABSIs.

Though the Infectious Diseases Society of America (IDSA) CLABSI guideline updates are in progress, our study adds to the body of evidence suggesting that for *Enterococcus* CLABSIs,^[24]^ catheter removal should be a focus of therapy, as it was not mandated in previous guidelines.^[5]^

Our study has several limitations. The retrospective nature of the study makes it difficult to elucidate possible confounders that could have biased the outcome measures. It is possible that patients with short- and long-term CVCs have different mortality rates when CVCs are not removed after MDRO CLABSI. However, it was impossible for us to retrospectively determine the type of CVC in each patient due to limitations of documentation in our electronic medical record system. In addition, the retrospective design of our study makes it impossible to determine why CVCs were left in place. We attempted to determine the reasons that CVCs remained in place for a random sample of 10% of the patients. During this review, we were unable to find any documentation as to why the CVC was left in place due to one of 3 reasons: CVC was not discussed, notes were illegible and no assessment was possible, or notes from the time of catheter removal could not be retrieved.

This was a single-center study and results may not be generalizable to other centers. Our cohort was predominantly white, middle-aged males, which may not be generalizable to other centers. However, patients more likely to need CVCs such as those receiving stem cell transplants or on dialysis are more likely to be older, white males, so our results may applicable in many settings with transplant and dialysis centers.^[27,28]^ The strength of the association between failure to remove a CVC and mortality is robust and likely applicable to other tertiary-care referral centers with similar patient case mixes. Another strength of our study is the size of the cohort, 430 patients with MDRO CLABSIs. In previous studies from our institution, we have shown high rates of drug resistance for various infections, ranging from 20% to 33%.^[29–32]^

As MDROs are more difficult to treat,^[6]^ source control with catheter removal would, in theory, be more critical for patients with MDRO CLABSIs, but we are limited by a lack of data on non-MDRO pathogens. Previous work has shown that patients with gram-negative MDRO CLABSIs fare worse when their catheters are not removed.^[25]^ Therefore, we felt it was more useful to include different types of MDROs rather than comparing to patients with non-MDRO infections. In addition, there were differences between patients that had their catheter removed and those that did not. We tried to account for this by including these factors in our multivariate Cox-proportional hazards model.

Another limitation of our study is the unavailability of antibiotic treatment data. Inappropriate antimicrobial therapy is a known risk factor for mortality in patients with bloodstream infections^[33]^ and this could affect the hazards ratios for death that we calculated in our Cox proportional hazards model. However, the strength of association between failure to remove a CVC and mortality is strong enough that it is unlikely that addition of antibiotic treatment data would negate this risk factor. Future studies should incorporate antibiotic treatment data into their multivariate models to definitively address this limitation.

In conclusion, failure to remove a CVC for patients with MDRO CLABSIs is associated with increased 30-day all-cause mortality. Comorbidities and requiring ICU care also were risk factors for mortality. By analyzing outcomes after MDRO CLABSI, we hope to encourage all providers to remove CVCs whenever a MDRO CLABSI is identified.

## Author contributions

**Conceptualization:** Jason P. Burnham, Marin H. Kollef.

**Data curation:** Jason P. Burnham, Rebecca P. Rojek, Marin H. Kollef.

**Formal analysis:** Jason P. Burnham, Marin H. Kollef.

**Investigation:** Jason P. Burnham, Rebecca P. Rojek, Marin H. Kollef.

**Methodology:** Jason P. Burnham, Rebecca P. Rojek, Marin H. Kollef.

**Project administration:** Jason P. Burnham, Marin H. Kollef.

**Resources:** Jason P. Burnham, Marin H. Kollef.

**Supervision:** Marin H. Kollef.

**Validation:** Jason P. Burnham, Marin H. Kollef.

**Visualization:** Jason P. Burnham, Marin H. Kollef.

**Writing – original draft:** Jason P. Burnham, Rebecca P. Rojek, Marin H. Kollef.

**Writing – review & editing:** Jason P. Burnham, Rebecca P. Rojek, Marin H. Kollef.

Jason P. Burnham orcid: 0000-0002-4777-3006.

## References

[1] CDC. Data Tables (Updated March 2016). In: HAI-Progress-Tables.xlsx. Available at: https://www.cdc.gov/hai/surveillance/progress-report/index.html, CDC, 2018 Accessed March 14, 2018.

[2] Rosenthal VD Maki DG Mehta Y International Nosocomial Infection Control Consortium (INICC) report, data summary of 43 countries for 2007–2012. Device-associated module. Am J Infect Control 2014;42:942–56. 25179325 10.1016/j.ajic.2014.05.029

[3] Klevens RM Edwards JR Richards CL Jr Estimating health care-associated infections and deaths in U.S. hospitals, 2002. Public Health Rep 2007;122:160–6. 17357358 10.1177/003335490712200205 PMC1820440

[4] Scott RD The Direct Medical Costs of Healthcare-Associated Infections in U.S. Hospitals and the Benefits of Prevention. Atlanta: Centers for Disease Control and Prevention, 2009. http://www.cdc.gov/hai/pdfs/hai/scott_costpaper.pdf Accessed March 14, 2018.

[5] Mermel LA Allon M Bouza E Clinical practice guidelines for the diagnosis and management of intravascular catheter-related infection: 2009 Update by the Infectious Diseases Society of America. Clin Infect Dis 2009;49:1–45. 19489710 10.1086/599376 PMC4039170

[6] Centers for Disease Control and Prevention Antibiotic Resistance Threats in the United States, 2013. Atlanta, GA: Centers for Disease Control and Prevention; 2013.

[7] Weiner LM Webb AK Limbago B Antimicrobial-Resistant Pathogens Associated With Healthcare-Associated Infections: Summary of Data Reported to the National Healthcare Safety Network at the Centers for Disease Control and Prevention, 2011–2014. Infect Control Hosp Epidemiol 2016;37:1288–301. 27573805 10.1017/ice.2016.174 PMC6857725

[8] Kuo SH Lin WR Lin JY The epidemiology, antibiograms and predictors of mortality among critically-ill patients with central line-associated bloodstream infections. J Microbiol Immunol Infect 2018;51:401–10. 28943144 10.1016/j.jmii.2017.08.016

[9] Tedja R Gordon SM Fatica C The descriptive epidemiology of central line-associated bloodstream infection among patients in non-intensive care unit settings. Infect Control Hosp Epidemiol 2014;35:164–8. 24442079 10.1086/674856

[10] Kaur M Gupta V Gombar S Incidence, risk factors, microbiology of venous catheter associated bloodstream infections - a prospective study from a tertiary care hospital. Indian J Med Microbiol 2015;33:248–54. 25865976 10.4103/0255-0857.153572

[11] CDC. Unusual Susceptibility Profiles Alert. Available at: http://www.cdc.gov/nhsn/pdfs/gen-support/usp-alert-current.pdf Accessed July 19, 2016.

[12] Multidrug-Resistant Organism & Clostridium difficile Infection (MDRO/CDI) Module. Available at: http://www.cdc.gov/nhsn/PDFs/pscManual/12pscMDRO_CDADcurrent.pdf Accessed July 19, 2016.

[13] Magiorakos AP Srinivasan A Carey RB Multidrug-resistant, extensively drug-resistant and pandrug-resistant bacteria: an international expert proposal for interim standard definitions for acquired resistance. Clin Microbiol Infect 2012;18:268–81. 21793988 10.1111/j.1469-0691.2011.03570.x

[14] Bloodstream Infection Event (Central Line-Associated Bloodstream Infection and Non-central Line Associated Bloodstream Infection). Centers for Disease Control and Prevention website. https://www.cdc.gov/nhsn/PDFs/pscManual/4PSC_CLABScurrent.pdf

[15] Harris PS Stalam T Ache KA Can hospices predict which patients will die within six months? J Palliative Med 2014;17:894–8. 10.1089/jpm.2013.0631 PMC4118712 24922330

[16] Teno JM Plotzke M Gozalo P A national study of live discharges from hospice. J Palliative Med 2014;17:1121–7. 10.1089/jpm.2013.0595 25101752

[17] CLSI. Performance Standards for Antimicrobial Susceptibility Testing; Twenty-Fifth Informational Supplement. CLSI document M100-S25 Vol. 25. Wayne, PA: Clinical and Laboratory Standards Institute, 2015.

[18] Dortet L Cuzon G Ponties V Trends in carbapenemase-producing Enterobacteriaceae, France, 2012 to 2014. Euro Surveill 2017;22: 10.2807/1560-7917.ES.2017.22.6.30461 PMC5316908 28205502

[19] Chang YT Coombs G Ling T Epidemiology and trends in the antibiotic susceptibilities of Gram-negative bacilli isolated from patients with intra-abdominal infections in the Asia-Pacific region. Int J Antimicrob Agents 2017;49:734–9. 28435019 10.1016/j.ijantimicag.2017.01.030

[20] Tansarli GS Karageorgopoulos DE Kapaskelis A Impact of antimicrobial multidrug resistance on inpatient care cost: an evaluation of the evidence. Expert Rev Anti Infect Ther 2013;11:321–31. 23458771 10.1586/eri.13.4

[21] Burnham JPKJ Olsen MA Babcock HM Readmissions with multidrug resistant infection in patients with prior multidrug resistant infection. Infect Control Hosp Epidemiol 2018;39:12–9. 29248023 10.1017/ice.2017.254 PMC6233291

[22] Burnham JP Olsen MA Stwalley D Infectious diseases consultation reduces 30-day and 1-year all-cause mortality for multidrug-resistant organism infections. Open Forum Infect Dis 2018;5:ofy026. 29577058 10.1093/ofid/ofy026 PMC5852998

[23] Fowler VG Jr Justice A Moore C Risk factors for hematogenous complications of intravascular catheter-associated *Staphylococcus aureus* bacteremia. Clin Infect Dis 2005;40:695–703. 15714415 10.1086/427806

[24] Marschall J Piccirillo ML Fraser VJ Catheter removal versus retention in the management of catheter-associated enterococcal bloodstream infections. Can J Infect Dis Med Microbiol 2013;24:e83–7. 24421837 10.1155/2013/678503 PMC3852445

[25] Lee YM Moon C Kim YJ Clinical impact of delayed catheter removal for patients with central-venous-catheter-related Gram-negative bacteraemia. J Hosp Infect 2018;99:106–13. 29330016 10.1016/j.jhin.2018.01.004

[26] Lee Y Lee YT Wang YC Risk of mortality of catheter-related bloodstream infections caused by *Acinetobacter* species: is early removal of the catheters associated with a better survival outcome? J Intensive Care Med 2018;33:361–9. 27872410 10.1177/0885066616677710

[27] Choi AI Rodriguez RA Bacchetti P White/black racial differences in risk of end-stage renal disease and death. Am J Med 2009;122:672–8. 19559170 10.1016/j.amjmed.2008.11.021 PMC2749005

[28] Mielcarek M Gooley T Martin PJ Effects of race on survival after stem cell transplantation. Biol Blood Marrow Transplant 2005;11:231–9. 15744242 10.1016/j.bbmt.2004.12.327

[29] Vazquez-Guillamet C Scolari M Zilberberg MD Using the number needed to treat to assess appropriate antimicrobial therapy as a determinant of outcome in severe sepsis and septic shock. Crit Care Med 2014;42:2342–9. 25072764 10.1097/CCM.0000000000000516

[30] Dallas J Skrupky L Abebe N Ventilator-associated tracheobronchitis in a mixed surgical and medical ICU population. Chest 2011;139:513–8. 20724738 10.1378/chest.10-1336

[31] Fisher K Trupka T Micek ST A prospective one-year microbiologic survey of combined pneumonia and respiratory failure. Surg Infect 2017;18:827–33. 10.1089/sur.2017.111 28880805

[32] Labelle A Juang P Reichley R The determinants of hospital mortality among patients with septic shock receiving appropriate initial antibiotic treatment. Crit Care Med 2012;40:2016–21. 22584765 10.1097/CCM.0b013e318250aa72

[33] Kollef MH Sherman G Ward S Inadequate antimicrobial treatment of infections: a risk factor for hospital mortality among critically ill patients. Chest 1999;115:462–74. 10027448 10.1378/chest.115.2.462

